# New Step in Understanding the Pathogenetic Mechanism of Sudden Infant Death Syndrome: Involvement of the Pontine Reticular Gigantocellular Nucleus

**DOI:** 10.3390/ijms25136920

**Published:** 2024-06-25

**Authors:** Anna Maria Lavezzi, Riffat Mehboob, Francesco Piscioli, Teresa Pusiol

**Affiliations:** 1 “Lino Rossi” Research Center for the Study and Prevention of Unexpected Perinatal Death and SIDS, Department of Biomedical, Surgical and Dental Sciences, University of Milan, 20122 Milano, Italy; 2 Lahore Medical Research Center, Lahore 54000, Pakistan; mehboob.riffat@gmail.com; 3 National Heart Lung and Blood Institute, National Institute of Health, Bethesda, MD 20892, USA; 4 Provincial Health Care Services, Institute of Pathology, Santa Maria del Carmine Hospital, 38068 Rovereto, Italy; francesco.pisciori@gmail.com (F.P.); teresa.pusiol@apss.tn.it (T.P.)

**Keywords:** neuropathology, brainstem, SIDS, gigantocellular nucleus, maternal smoking

## Abstract

This study aimed to investigate, for the first time, the potential role of the gigantocellular nucleus, a component of the reticular formation, in the pathogenetic mechanism of Sudden Infant Death Syndrome (SIDS), an event frequently ascribed to failure to arouse from sleep. This research was motivated by previous experimental studies demonstrating the gigantocellular nucleus involvement in regulating the sleep–wake cycle. We analyzed the brains of 48 infants who died suddenly within the first 7 months of life, including 28 SIDS cases and 20 controls. All brains underwent a thorough histological and immunohistochemical examination, focusing specifically on the gigantocellular nucleus. This examination aimed to characterize its developmental cytoarchitecture and tyrosine hydroxylase expression, with particular attention to potential associations with SIDS risk factors. In 68% of SIDS cases, but never in controls, we observed hypoplasia of the pontine portion of the gigantocellular nucleus. Alterations in the catecholaminergic system were present in 61% of SIDS cases but only in 10% of controls. A strong correlation was observed between these findings and maternal smoking in SIDS cases when compared with controls. In conclusion we believe that this study sheds new light on the pathogenetic processes underlying SIDS, particularly in cases associated with maternal smoking during pregnancy.

## 1. Introduction

Sudden Infant Death Syndrome (SIDS) is the leading cause of death for infants under one year of age, primarily occurring during the awakening phase from sleep. Worldwide SIDS is defined as a death that remains unexplained after a thorough investigation (including the performance of a complete autopsy, examination of the death scene, and review of the clinical history) [1]. 

Neuropathological research has long focused on investigating developmental alterations in various nuclei and structures of the human brain in SIDS, frequently highlighting the hypoplasia of various structures involved in breathing and sleep–wake control [2,3,4,5,6,7,8,9]. Anomalies of other nuclei not directly involved in these functions (but that widely project to nuclei containing respiratory neurons) have also been detected in this syndrome, such as hypoplasia of the raphe nuclei, which are components of the reticular formation (RF) [10,11]. The RF is a vast network of neurons and nerve fibers distributed along the brainstem midline, subdivided in nuclei without a precise delineation of each other [12,13,14]. These RF nuclei are able to connect and coordinate many brain structures of vital importance by releasing specific neurotransmitters [15,16]. 

Despite its widespread structure, the RF network has been subdivided into three longitudinal columns: the “median column” that contains the raphe nuclei, which are distributed along the median brainstem region, the “medial column”, grouping the so-called gigantocellular nuclei (due the presence of larger neurons) extended from the medulla oblongata to the pons medially to the median column, and the “lateral column”, mainly composed of parvocellular nuclei (because of the small size of the cells) distributed laterally to the median zone [12,13,14]. Nevertheless, except for the previously mentioned studies concerning the raphe nuclei, no developmental anomalies of other components of the RF have yet been highlighted in SIDS. 

In this regard, this paper intends to deepen the investigations into a group of SIDS cases that mainly occurred during sleep in order to evaluate whether, in addition to the brainstem respiratory center, the gigantocellular nucleus (GCn), which is the main component of the medial RF portion, may be involved in these deaths. This research was motivated by previous experimental studies demonstrating the GCn’s involvement in regulating the sleep–wake cycle [17,18,19,20,21,22]. In the context of this study, we also aim to explore the potential role of the GCn in regulating catecholamine synthesis via tyrosine hydroxylase (TH), as imbalances of this enzyme have frequently been documented in SIDS [23,24,25,26]. Furthermore, an additional goal is to investigate whether any developmental alterations in the GCN can be linked to the mother’s medical history.

## 2. Results

In line with the purpose of this study, the neuropathological examination was mainly focused on the reticular GCn in a series of 28 SIDS and 20 control cases. In brainstem cross sections, this nucleus exhibits a roughly quadrangular structure in outline throughout its extent, which is smaller in the medulla oblongata and more extensive in the caudal pons. Most GCn neurons are large, multipolar, and darkly stained due to the presence of Nissl granules, which appear as irregular aggregates mainly located at the peripheral zone of the cytoplasm. The nucleus often assumes an eccentric position. Smaller cells, always rich in Nissl granules, are often mixed with these neurons. 

Although no significant cytoarchitectural or functional anomalies were observed in the medullary portion of the GCn of either the SIDS cases or the controls, morphological and immunohistochemical differences were detected in its upper part (caudal pons). For this reason, only the results relevant to the rostral portion of this nucleus, henceforth indicated with the acronym “pGCn”, where “p” means “pontine”, are included below. 

### 2.1. Morphological Examination of the pGCn

**Figure 1:**
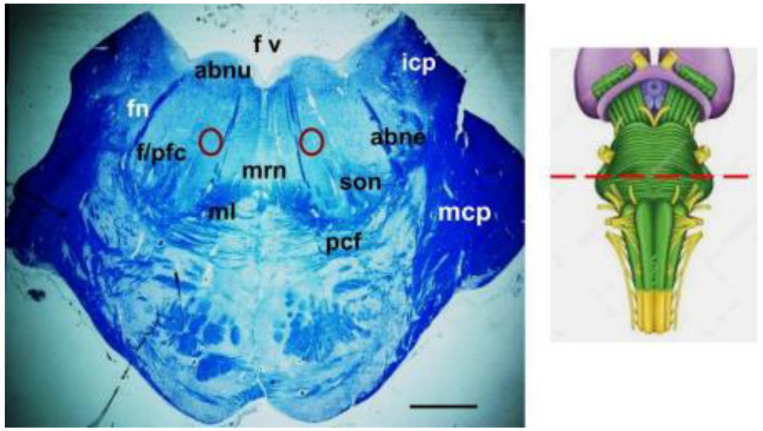
Transversal section of the caudal pons at the level where the abducens nucleus, the facial/parafacial complex, and the superior olivary nucleus are visible. The red circles indicate the location of the pontine gigantocellular nucleus. Klüver–Barrera staining. Scale bar 1500 μm. Abne = abducens nerve; abnu = abducens nucleus; fn = facial nerve; f/pfc = facial/parafacial complex; fv = fourth ventricle; icp = inferior cerebellar peduncle; mcp = middle cerebellar peduncle; ml = medial lemniscus; mrn = magnus raphe nucleus; pcf = pontocerebellar fibers; son = superior olivary nucleus. Top right: Brainstem diagram with indication (red dashed line) of the pontine level of the section represented here.

The histological examination of the reticular pGCn was performed on specific serial sections, at the same pontine levels for both SIDS cases and controls, by comparing the results obtained for each SIDS case with those of the age-matched controls. Landmarks of these sections are represented by the presence in each of them of the abducens nucleus, the facial/parafacial complex, and the superior olivary nucleus (Figure 1). 

**Figure 2:**
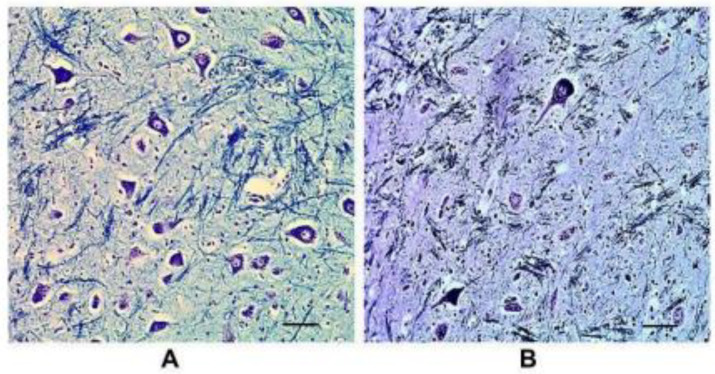
(**A**) pGCn in histological sections of caudal pons. (**A**) Normal structure of the pGC (control case—male, 4 months); (**B**) hypoplasia of pGCn with low number of cells in a SIDS case (male, 4 months). Klüver–Barrera staining. Scale bar 70 μm.

The overall results obtained from the cytoarchitectural analysis of the pGCn led to the following conclusions:

- The pGCn showed the same widespread distribution of many neurons in all controls and nine SIDS cases (Figure 2A). This finding was considered to be in line with normal nucleus development for infants under one year of age.
- The pGCn outlook differed for the remaining 19 SIDS cases due to the presence of rare (sometimes extremely rare) neurons. A diagnosis of “pGCn hypoplasia” was made for these cases (Figure 2B).

### 2.2. TH—Immunohistochemical Examination of the pGCn 

**Figure 3:**
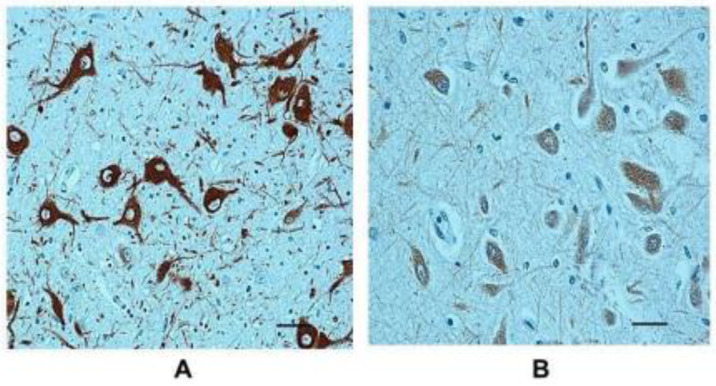
Photomicrographs showing (**A**) tyrosine hydroxylase-positive immunolabeled cells in the pGCn of a control case (female, 3 months) and (**B**) tyrosine hydroxylase-negative cells in a SIDS case with a normal GCn structure (female, 3 months). TH immunohistochemistry. Scale bar, 100 μm.

Sections immunolabeled for TH showed intensely positive neurons in the pGCn (>80% of neurons) in almost all controls, more specifically in 18 control cases, and in 11 SIDS cases (7 of which with pGCn hypoplasia and 4 with normal pGCn structure) (Figure 3A). In the remaining 17 SIDS cases (12 with pGCn hypoplasia and 5 with normal pGCn structure) and 2 controls, TH immunopositivity was weak or not expressed (<10% of neurons) (Figure 3B). 

Overall, SIDS cases showed a significantly higher incidence of histological/immunohistochemical alterations in the pGCn than the age-matched controls. In particular, developmental structural defects (hypoplasia) of the pGCn were observed in 17 SIDS cases, but in no control cases. Similarly, low-to-complete absence of TH expression was found with significantly higher incidence rates in the SIDS group (17 cases) than the control group (2 cases). 

Table 1 summarizes both the morphological and immunohistochemical findings described above. 

### 2.3. Correlation of the Results with Risk Factors

#### 2.3.1. Correlations with Cigarette Smoke

Eighteen SIDS mothers and 6 control group mothers were either tobacco or e-cigarette smokers (more than three cigarettes per day) before pregnancy and after their child’s birth. 

A strong correlation was observed between pGCn hypoplasia, TH negative/low immunoexpression, and maternal smoking in SIDS cases compared with the control cohort (*p* < 0.01). Overall, 15 of the 19 SIDS cases with pGCn hypoplasia were born to one of the 18 mothers of SIDS infants who smoked. No or mild TH immunostaining was observed in a few of the neurons of 11 of these cases. The remaining three SIDS cases born to smoking mothers showed weak TH expression in pGCn neurons with a normal structure. 

Regarding the control group, TH expression deficiency was observed in two of the six cases born to smoking mothers, while no alterations were detected in the other four cases (Table 2). 

#### 2.3.2. Correlations with Alcohol/Drug Misuse and Environmental Pollutants 

No correlation was assessed regarding the intake of alcohol or drugs, as all mothers denied having taken these substances. Differences, although not significant, were detected between SIDS cases and controls regarding the exposure to airborne toxicants (such as particulate matter, pesticides, polycyclic aromatic hydrocarbons, toxic metals, etc.). Based on our investigations, only five mothers of the SIDS group (18%) and two mothers of the control group (10%) spent their pregnancy and the postpartum period in agricultural areas where pesticides are often used, or near industries that use toxic materials. 

## 3. Discussion

The focus of this study was on one of the components of the reticular formation, namely the gigantocellular nucleus (GCn), with the aim of broadening our knowledge on the pontine tract (pGCn) due to its involvement in the control of sleep–wake cycles, as highlighted in experimental studies. In particular, Goutagny et al. [18] proved that bilateral lesions of the pontine GC tegmental field in cats resulted in the complete elimination of paradoxical sleep (REM phase), a recurring part of the sleep–wake cycle. Similarly, Siegel et al. [19] demonstrated that pGC field neurons are able to modulate sleeping and waking activity. The study of Sirieix et al. [21] explained how the pGCn interacts with other brainstem nuclei, such as the locus coeruleus and raphe nuclei, to influence the sleep–wake cycle by initiating and maintaining wakefulness and helping to regulate rapid-eye-movement (REM) sleep. Consequently, damage or dysfunction of the pGCn may result in sleep disorders. 

In the context of this study, in addition to the morphological examination of the GCn with specific histological techniques, we applied TH immunohistochemistry, a valuable tool in neuropathology, as it specifically detects the presence of tyrosine hydroxylase, a key enzyme responsible for the synthesis of catecholamines (adrenaline, noradrenaline, and dopamine) in neurons [27,28]. A decrease in TH immunoreactivity is a common feature of neurodegenerative diseases like Parkinson’s disease, in which there is a reduction in dopamine production [29,30]. As there are currently no studies demonstrating how p GCn can affect SIDS, a syndrome that mostly occurs during sleep, especially upon awakening, we aimed to analyze the TH expression in the GCn in a series of babies who died suddenly, aligning with the aforementioned study by Gao et al. [17]. Notably, these authors identified a subset of neurons within the GCn that can promote arousal from sleep by activating the cholinergic, noradrenergic, and glutamatergic arousal pathways. 

The results of our study fulfilled our expectations as they highlighted a significantly higher incidence of both structural and functional neuronal abnormalities in the pontine portion of the GCn in SIDS cases than in the age-matched controls. More specifically, neuronal deficiency (hypoplasia, even severe) of the pGCn was found in a high percentage of SIDS cases (68%), contrary to what was observed in all control cases, in which the development of the pGCn constantly showed a normal structure. Similarly, the immunohistochemical expression of TH was low or absent in 61% of SIDS cases (12 of which with GCn hypoplasia), while this was observed in only 10% of control subjects. Normally, the TH level in the catecholaminergic neurons, and hence in the GCn, is highest during waking and decreases with oscillations during sleep [31]. Increases in TH proteins and mRNA have been reported to occur in normal subjects in response to stress conditions [32]. We therefore believe that, in most SIDS, given the low-to-complete absence of TH expression in pGCn neurons, this functional response does not occur by impairing the sleep–wake cycle. 

### 3.1. Genetics of the TH Enzyme

In humans, the TH enzyme is encoded by the TH gene, which contains a tetrameric short tandem repeat in intron 1 (TH01) [33,34]. Mutations or variants (polymorphisms, rare variants) of the TH gene can result in the reduced activity of TH enzymes, thus affecting normal nervous system function. 

A polymorphism of the TH gene, namely allele 9.3, has been investigated in several SIDS studies [35,36,37]. This genetic variant causes defects in the synthesis and/or storage of catecholamines, especially of noradrenaline, which is essential for regulating breathing and arousal. Our finding of poor or absent TH expression observed during the immunohistochemical examination of the pGCn, especially in SIDS, suggests the existence of this polymorphism. Of course, we intend to confirm our assumption by analyzing the TH genetic features of all the cases involved in our study as soon as possible. 

The significant association observed between abnormal findings (namely hypoplasia and TH-negative/low immunoexpression) and maternal smoking, particularly within the SIDS group, is noteworthy. 

Our observation of nicotine exposure in utero causing defects in TH expression and cytoarchitecture specifically in the pGCn, but not in other brainstem centers, differs from previous studies showing a significant correlation between maternal smoking during pregnancy and alterations in various brain regions [38,39,40,41,42]. This finding suggests biological variability in response to prenatal smoke exposure, impacting the development of specific structures in different brain areas. 

The negative effects of maternal smoking, especially during pregnancy, on the newborn have been widely documented by numerous studies worldwide. Among these, low birth weight (LBW) and small size for gestational age (SGA) are well known [43,44,45]. Furthermore, a large number of epidemiologic studies have shown a very high relationship between maternal smoking and SIDS [46,47,48,49,50]. 

Specific morphological and functional developmental alterations in the nervous system, such as serotonergic alterations, nicotinic receptor alterations, grow factor expression abnormalities, hypoplasia of brainstem nuclei, and alterations in the nerve centers involved in regulating cardiorespiratory function, have been found to be related to prenatal nicotine exposure [38,39,40,41,42,51,52,53]. When a mother smokes during pregnancy, the nicotine is rapidly absorbed by the lungs and enters the maternal bloodstream. From here, above all, the CO, a gaseous combustion product of nicotine, crosses the placental barrier and passes into the fetal bloodstream forming carboxyhemoglobin, a complex that creates hypoxic conditions in the tissues as it is unable to release oxygen. 

Furthermore, nicotine is one of the few lipid-soluble substances that can readily cross the blood–brain barrier. This allows it direct access to fetal brain parenchyma, where it can interfere with the expression of genes that control nervous system development. 

### 3.2. Limitations of the Study 

The study was limited by the difficulty we encountered in ensuring consistent and optimal fixation times for the brain samples in both the SIDS and control groups, as we are aware that prolonged fixation can affect immunohistochemical TH expression. Another limitation consists of the disparity between the number of SIDS cases and that of control cases (*n* = 28 SIDS vs. *n* = 20 controls). However, a smaller size of the control group is common in the literature in studies performed on SIDS, and this is indicative of the general difficulty in finding infants who died in the first months of life from well-defined causes. 

### 3.3. Conclusions

As a concluding remark, our findings suggest that pGCn developmental defects, encompassing both structural and functional abnormalities, alone or in association with other brain abnormalities, may contribute to impaired motor activation during awakening, a hallmark of SIDS. This effect might be particularly pronounced when combined with hypoxia caused by maternal cigarette smoking during pregnancy. We believe this study has the potential to reveal novel insights into the pathological mechanisms underlying SIDS, especially in cases with established risk factors like maternal smoking. 

## 4. Materials and Methods

### 4.1. Study Subjects

This study included a cohort of SIDS cases and a control group, comprising a total of 48 infants.

- The SIDS group was composed of 28 cases (12 females and 16 males, aged from 1 to 7 postnatal months). SIDS was diagnosed because the routine post-mortem examination was unable to establish any cause of death. Therefore, an in-depth analysis of the nervous system was carried out with particular investigation of the brainstem, where main nervous centers of vital importance are located, in accordance with the directives of Italian law n.31/2006 “Regulations for Diagnostic Post Mortem Investigation in Victims of Sudden Infant Death Syndrome (SIDS) and Unexpected Fetal Death” [54]. These 28 cases were selected from a large number of SIDS victims in which no developmental abnormalities in the main vital nervous centers were detected at the thorough neuropathological examination.
- The control group was composed of 20 suddenly deceased subjects (8 females and 12 males, aged from 2 to 7 postnatal months) for whom a complete autopsy and clinical history analysis established a precise cause of death. Specific diagnoses included the following: congenital heart disease (*n* = 6); respiratory infection (*n* = 6); disorders related to prematurity (*n* = 2); traumatic incident (*n* = 2); congenital malformations (*n* = 2); medium-chain acyl-coenzyme A dehydrogenase (MCAD) deficit (*n* = 1); sepsis (*n* = 1).

For each case, a complete clinical history, including all available data regarding the pregnancy, birth, and the environmental situation in which the death occurred, was collected. 

All the cases of this study came from Italian regions (mostly from Trentino), based on the directives of the aforementioned Italian law. 

### 4.2. Risk Factor Information

Information on the main potential risk factors (above all, maternal cigarette smoking, alcohol and drug misuse before and during pregnancy, and environmental risks present in the area where the pregnant mothers and their babies lived) were collected and analyzed. Particular emphasis was given to maternal cigarette (including e-cigarette) smoking. 

Based on their statements, 18 of the 28 SIDS mothers (64%) were active smokers (with consumption of more than 3 cigarettes/day) before, during, and after pregnancy until the child’s death. The remaining 10 mothers (36%) denied having smoked in the past. Little information was available on the smoking habits of the victims’ fathers or of other people living in the household. 

Six of the twenty control mothers (30%) admitted to smoking more than 3 cigarettes a day before their pregnancy. The remaining 14 mothers (70%) denied the habit of smoking.

No relevant information was obtained regarding maternal alcohol and/or drug misuse. Information on the presence of pollutants in the environment where the mother lived until her child’s death was obtained in a few cases (5 SIDS cases and 2 controls). 

### 4.3. Neuropathological Examination

The methods used for the in-depth examination of the nervous system are described in our previous articles [8,9,55]. 

The focus of this study was on the GCn. This nucleus extends from the upper third of the medulla oblongata—where it can be found dorsal to the inferior olivary nucleus and lateral to the raphe obscurus nucleus—to the caudal portion of the pons, where it reaches its maximum size. 

The portion of the brainstem where the GCn is located was first cut into transverse sequential slices orthogonally to the brainstem axis. Then, 5–6 μm sections were obtained from each of them. Two of these were stained for the histological examination of the GCn cytoarchitecture by using, in addition to the routine hematoxylin–eosin staining, the more specific Klüver–Barrera approach, while immunohistochemical assays were applied on other sections in order to determine the neuronal expression of tyrosine hydroxylase (TH), a marker of catecholaminergic neurons [27,28], in the GCn. 

#### TH (Tyrosine-Hydroxylase) Immunohistochemistry

The selected sections were rinsed three times in Trizma-buffered saline (TBS) 0.1 M, pH 7.4, and then incubated at 4 °C in a 1/500 dilution of primary rabbit antiserum to TH (Chemicon, Temecula, CA, USA) for 48 h. The dilutions were prepared with a solution of 1% normal goat serum (NGS) in 0.05 TBS. This was followed by a 2.5 h incubation in a 1/200 dilution of biotinylated goat anti-mouse immunoglobulin G (IgG) (Vector Laboratories, Burlingame, CA, USA) in 1% NGS in Tris saline. The sections were then incubated for 2 h with the avidin–biotin complex (Vector Laboratories) diluted 1/100 with 1% NGS in Tris saline. Between each incubation, the sections were rinsed three times with 1% NGS in Tris saline. The sections were then treated for 6 min with a 0.05% solution of 3,3′diamino-benzidine and 0.01% hydrogen peroxide, rinsed in phosphate buffer, mounted on gel-coated slides, cleared in xylene, and coverslipped with Depex mounting medium. 

All of the observations were acquired with a Nikon Eclipse E800 light microscope (Nikon Corporation, Tokyo, Japan) and the images of interest were taken using a Nikon Coolpix 8400 digital camera (Nikon Corporation, Tokyo, Japan) attached to the microscope. 

### 4.4. Statistical Methods

All findings were analyzed by two blinded independent pathologists. The evaluations of each observer regarding the various parameters were reported separately in a case report table. After the mean values were calculated, they were compared using the K Index (KI) in order to determine interobserver reproducibility. The Landis and Koch [56] method was then used to interpret the K values (0 to 0.2 = slight agreement; 0.21 to 0.40 = fair agreement; 0.41 to 0.60 = moderate agreement; 0.61 to 0.80 = strong or substantial agreement; 0.81 to 0.99 = very strong or almost perfect agreement; 1.0 = perfect agreement). A very satisfactory KI value (0.89) was obtained in this study. The statistical significance of the direct comparisons between groups was determined using the analysis of variance. The calculations were performed with SPSS software (statistical package for social science, version 11.0; SPSS Inc., Chicago, IL, USA). Differences were considered statistically significant if the *p* values were <0.05. 

### 4.5. Ethics Approval and Consent

Institutional review board approval was not required for this study since it complies with the requirements of the aforementioned Italian law. Regardless, parents of all SIDS cases and all controls provided written informed consent to both autopsy and related research. 

## Figures and Tables

**Table 1:** pGCn morphological and immunohistochemical results of SIDS and control cases.

	SIDS n.28		Controls n.20
**pGCn** **cytoarchitecture**	**Normal**	9 (32%)	20 (100%)
	**Hypoplasia**	19 (68%)	0 (0%)
**pGCn** **TH immunoexpression**	**Normal**	11 * (39%)	18 (90%)
	**Weak/negative**	17 ** (61%)	2 (10%)

Categorical data are expressed as number of cases and percentages. * Four of these cases with normal pGCn structure and seven with pGCn hypoplasia. ** Five of these with pGCn normal structure and twelve with pGCn hypoplasia. Statistical significance of SIDS vs. control group values *p* < 0.01. pGCn: pontine gigantocellular nucleus; TH: tyrosine hydroxylase.

**Table 2:** Correlation between maternal smoking and pGCn morphological/immunohistochemical results in SIDS and controls.

	SIDS		Controls	
**Maternal smoking**	**Yes**	**No**	**Yes**	**No**
	**18** (64%)	**10** (36%)	**6** (30%)	**14** (70%)
**Findings**				
Normal pGCn cytoarchitecture + normal TH expression	0	10	4	14
pGCn hypoplasia *	15	0	0	0
Normal pGCn cytoarchitecture and TH weak/negative expression	3	0	2	0

Categorical data are expressed as number of cases. * Twelve of these cases with weak TH expression. Statistical significance of SIDS vs. control group values in relation to maternal smoking *p* < 0.01 pGCn: pontine gigantocellular nucleus; TH: tyrosine hydroxylase.

## Data Availability

The data presented in this study are available on request from the corresponding author.
